# VMESR: Variable Mamba-Enhanced Super-Resolution for Real-Time Road Scene Understanding with Automotive Vision Sensors

**DOI:** 10.3390/s26051683

**Published:** 2026-03-06

**Authors:** Hongjun Zhu, Wanjun Wang, Chunyan Ma, Rongtao Hou

**Affiliations:** 1 School of Computer and Software Engineering, Anhui Institute of Information Technology, Wuhu 241199, China; hjzhu3@iflytek.com (H.Z.); cyma@hhu.edu.cn (C.M.); rthou@iflytek.com (R.H.); 2 Institute of EduInfo Science and Technology, Nanjing Normal University, Nanjing 210024, China; 3 School of Artificial Intelligence, Hohai University, Nanjing 211100, China; 4 School of Computer Science, Nanjing University of Information Science and Technology, Nanjing 210044, China

**Keywords:** automotive vision systems, super-resolution, multi-scale feature extractor, feature fusion, automotive sensors, autonomous driving

## Abstract

Automotive vision systems depend critically on front-view cameras, whose image quality frequently degrades under adverse conditions such as rain, fog, low illumination, and rapid motion. To address this challenge, we propose VMESR, a variable mamba-enhanced super-resolution network that integrates a selective state-space model into a lightweight super-resolution architecture. By serializing 2D feature maps and applying variable-depth mamba blocks, VMESR captures long-range dependencies with linear complexity. A multi-scale feature extractor, enhanced residual modules equipped with a convolutional block attention module, and dense fusion connections work together to improve the recovery of high-frequency details. Extensive experiments demonstrate that VMESR achieves competitive performance in both objective metrics and perceptual quality compared to state-of-the-art methods, while significantly reducing parameter counts and computational cost. VMESR provides a practical balance between efficiency and reconstructive accuracy, offering a deployable super-resolution solution for embedded automotive sensors and enhancing the robustness of autonomous driving perception pipelines.

## 1. Introduction

Autonomous driving technology has advanced rapidly in recent years, becoming an integral part of future transportation systems. However, achieving full autonomy and ensuring its safety in complex and variable real-world road environments places critical demands on the perception capabilities of autonomous driving systems [1]. The vehicle’s visual perception system, particularly the front-view camera, is tasked with acquiring crucial information from the surrounding environment. By capturing image data, the front-view camera provides the necessary field of view for the autonomous driving system, aiding in the identification of various important targets such as traffic sign detection [2]. Nevertheless, the complexity and variability of actual driving conditions, especially under harsh weather, low light, and fast motion, pose severe challenges to the quality of visual data.

Existing image super-resolution techniques can be broadly categorized into traditional methods and deep learning-based methods [3]. Traditional methods such as interpolation algorithms are simple to implement but struggle to restore high frequency details in complex scenes and cannot handle long-range dependencies within images. For complex road scenes, traditional methods fail to effectively extract key detailed information, resulting in poor reconstruction [4]. While deep learning methods can extract complex image features by learning from vast amounts of data and reconstruct image details reasonably well, they typically require substantial computational resources and longer processing times, making them difficult to deploy on real-time, resource-constrained vehicular platforms [5]. Especially under the hardware and energy efficiency constraints of vehicular computing platforms, the high computational complexity of deep learning methods often fails to meet practical real-time requirements, thus falling short of the high standards for image quality demanded by autonomous driving systems [6].

**Figure 1:**
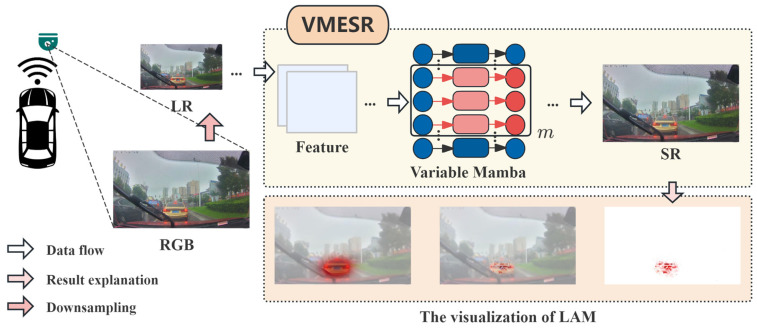
Graphical abstract of VMESR. The real-road condition RGB images captured by the on-board camera are processed through interpolation to obtain LR. VMESR reconstructs the LR images into SR images, and the local attribution map is used to visually evaluate the performance of VMESR.

We propose VMESR, a lightweight super-resolution architecture based on the selective state-space model for autonomous driving perception systems. This framework can significantly enhance the image quality of front-view cameras under challenging conditions, providing more reliable visual input for downstream critical perception tasks such as traffic sign recognition, pedestrian detection, and lane segmentation [7]. Its lightweight nature makes it suitable for deployment on resource-constrained embedded vehicular platforms, meeting the stringent real-time requirements of autonomous driving systems. This technology not only improves the performance of existing advanced driver assistance systems (ADAS), but also provides crucial technical support for the safety and reliability of next-generation fully autonomous driving systems, holding significant industrial application value and traffic safety implications. The graphical abstract of VMESR is shown in Figure 1. Our major contributions are as follows:

- We design a novel lightweight network architecture that introduces the mamba framework from state space models (SSMs) into the image super-resolution task. This architecture captures long-range dependencies via mamba’s selective scanning mechanism by transforming 2D image features into sequences while maintaining linear computational complexity.
- We design a multi-level feature enhancement mechanism, including a multi-scale feature extraction module, an enhanced residual (ER) module incorporating attention mechanisms, and dense residual connections, forming a multi-level feature enhancement mechanism that effectively improves the model’s ability to recover image details and textures.
- We apply this model to image super-resolution for automotive front-view cameras, which can enhance image quality under challenging conditions such as harsh weather and low illumination, and thus provide more reliable visual input for autonomous driving perception tasks (such as traffic sign recognition and pedestrian detection).

The remainder of this paper is organized as follows: Section 2 reviews related work. Section 3 details the proposed method, including the framework and modules. Section 4 presents the experimental setup, comparative experiments, and real-world application validation. Section 5 concludes the work.

## 2. Related Work

### 2.1. Single Image Super-Resolution

Single Image Super-Resolution (SISR) is a significant task in the field of image processing, aiming to reconstruct a high-resolution image from a low-resolution input. Early SISR methods typically relied on interpolation algorithms, such as bilinear and bicubic interpolation, or reconstruction techniques. While simple, these methods often fail to restore high frequency details in images, resulting in reconstructed outputs lacking sharpness and realism [8].

With the advancement of deep learning, Convolutional Neural Network (CNN)-based super-resolution methods have achieved remarkable progress. SRCNN [9] first introduced deep learning to the super-resolution (SR) task by training a convolutional network to recover high-resolution images from low-resolution inputs. Subsequently, methods like VDSR [10] and EDSR [11] further improved reconstructed image quality by employing deeper network architectures and more complex loss functions. However, these CNN-based methods generally cannot effectively capture long-range contextual information, which is crucial for SR reconstruction in complex scenes.

In recent years, the transformer architecture has also been introduced to SR tasks. Kang et al. [12] proposed an efficient Swin Transformer network (ESTNet) based on channel attention mechanisms, achieving superior SR reconstruction by combining efficient channel attention with grouped attention modules. Zhang et al. [13] designed a cascaded visual attention network (CVANet) that simulates the human visual attention mechanism to address SISR. This method adaptively reinforces key information through collaborative feature, channel, and pixel-level attention modules to enhance detail reconstruction. Zheng et al. [14] proposed an efficient mixed transformer (EMT) for SISR. This method introduces a pixel mixer to enhance local feature aggregation and employs a stripe window self-attention mechanism to establish global dependencies at a lower computational cost. Wang et al. [15] proposed a multi-scale attention network (MAN) for SR tasks, aiming to unleash the potential of ConvNets. By combining multi-scale mechanisms with large kernel attention, the network designs two modules—multi-scale large kernel attention and gated spatial attention units—to efficiently aggregate global and local information. Talreja et al. [16] effectively fused global dependencies with local features by incorporating local feature window transformers (LWFTs) to improve image quality. Xie et al. [17] proposed a model combining dilated convolutions with self-attention mechanisms, efficiently capturing local and global features through multi-range attention and sparse multi-range attention modules. These transformer-based methods model long-range dependencies in images through self-attention mechanisms, thereby improving image reconstruction quality. While these methods have made significant strides in various aspects of image restoration, their high computational complexity makes them difficult to apply in resource-constrained environments, such as embedded vehicular platforms.

### 2.2. Super-Resolution Applications in Automotive Vision Sensors

Automotive vision sensors play a crucial role in autonomous driving systems, with the front-view camera being particularly vital for tasks like obstacle detection, traffic sign recognition, and pedestrian detection [18]. However, the challenges faced by automotive vision sensors far exceed those of general image SR tasks, primarily manifesting as image degradation under adverse weather conditions, such as rain, snow, fog and low light environments [19].

Some research efforts are dedicated to improving image quality for vehicular sensors under these challenging conditions. For instance, deep learning-based SR methods are widely applied to enhance images from vehicle-mounted cameras. Shi et al. [20] proposed a generative adversarial network (GAN)-based SR method integrated into an object detection task, addressing issues like indistinct targets in blurred wildlife images captured by vehicle-mounted mobile monitoring systems. Premachandra et al. [21] proposed upsampling distant road areas in images by estimating the vanishing point of the road to locate the region requiring enhancement and specifically applying upsampling to this area to improve vehicle visibility, thereby enhancing vehicle detection accuracy in both day and night environments. To address challenges such as vehicle decoration differences, nonuniform license plate locations, and character occlusion, Usama et al. [22] proposed an automatic toll collection framework for complex scenes, realized through three steps: vehicle type recognition, license plate localization, and character reading. To resolve the contradiction between large field-of-view (FoV) images having low resolution and traditional images having high resolution but small FoV, Dong et al. [23] proposed enhancing resolution by embedding high-resolution images into large FoV images. Utilizing techniques based on nonrigid transformation and grid optimization, this method avoids correcting radial distortion in the large FoV image and preserves the shape and content of the original image by modeling the transformation relationship between images and optimizing control point distribution.

However, most existing methods overlook the real-time requirements of vehicular sensor systems [24]. Especially in real-time autonomous driving systems, where computational resources are limited and low latency is demanded, existing methods are difficult to deploy on embedded platforms. Therefore, SR methods for resource-constrained environments, particularly efficient algorithms satisfying real-time needs, remain an urgent problem to be solved.

### 2.3. State Space Models

State space models (SSMs) are methods for modeling time-series data by describing the dynamic relationships between system states and observations [25]. In recent years, selective state space models have been introduced into image processing tasks, becoming an important research direction in the field of image super-resolution due to their efficiency in capturing long-range dependencies [26].

Mamba is an innovative architecture based on SSMs, capturing long-range dependencies by serializing image features and applying a selective scanning mechanism [27]. Mamba possesses linear computational complexity, enabling it to maintain high performance while significantly reducing computational resource consumption. The mamba approach has achieved success in fields such as speech recognition and natural language processing [28]. Recently, mamba has been introduced into SR tasks to improve image restoration accuracy and efficiency. By serializing image features and applying variable mamba blocks, mamba can model global contextual information in images at low computational complexity, thereby effectively restoring image details and textures. Lei et al. [29] proposed a lightweight image SR network that combines visual mamba with a distillation strategy, maintaining performance while significantly reducing model parameters. Li et al. [30] proposed a hierarchical attention mamba network, achieving excellent reconstruction results through the innovative design of hierarchical aggregation attention modules and spatial frequency information interaction modules. Wang et al. [31] proposed a lightweight collaborative network of mamba and CNN. By combining the global receptive field of mamba with the local inductive bias of CNN and introducing a multi-scale spatial refinement attention mechanism, this network effectively enhanced feature representation capabilities. Zhang et al. [32] proposed a lightweight network mixing transformer and mamba. By alternately using transformer and mamba modules to reduce computation and designing transformer aggregation block (TAB) and mamba aggregation block (MAB) modules to enhance local and global feature extraction capabilities, combined with a proposed re-parameterized spatial gate feed-forward network (RepSGFN) component, high-resolution images were obtained.

Compared to traditional convolutional networks and transformer models, mamba demonstrates unique advantages in SR tasks. Particularly in automotive vision sensors, mamba can assist in achieving real-time SR reconstruction in computationally constrained environments through its efficient contextual modeling capability.

Existing image SR methods have made significant progress in improving image quality, especially techniques based on deep learning and transformers. However, the application of most methods in resource-constrained environments like automotive vision sensors remains challenging. Although existing research has attempted to combine lightweight networks with feature enhancement techniques, finding the optimal balance between computational efficiency and image quality is still an urgent problem. The proposed VMESR addresses this issue by introducing the selective state space model, combined with multi-level feature enhancement and attention mechanisms, offering a novel and efficient solution.

## 3. Methodology

**Figure 2:**
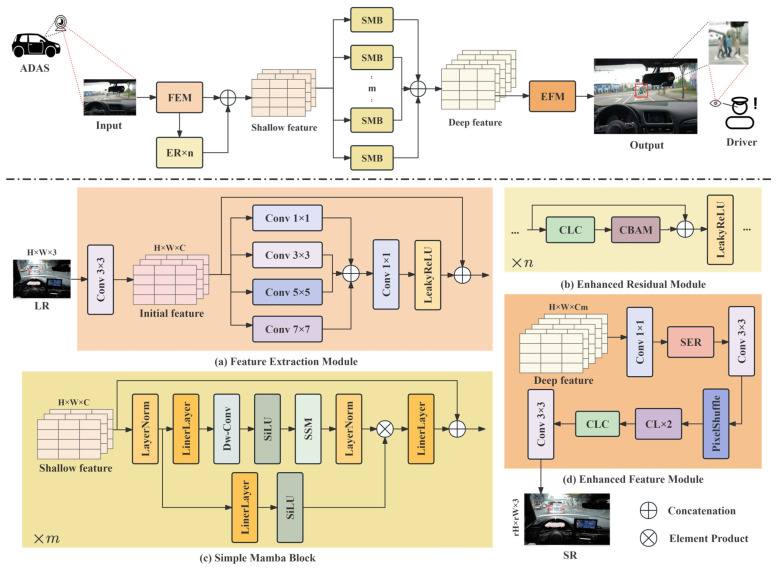
Illustrates the VMESR architecture, consisting of: (**a**) a feature extraction module that maps the input image from three channels to a high-dimensional feature space using convolutions of multiple scales; (**b**) an ER module that extracts and fuses enhanced shallow features through multiple enhanced residual blocks with attention mechanisms; (**c**) a variable mamba integration module composed of multiple single mamba blocks, which serializes features, captures long-range dependencies using the state space model, and fuses the output features of multiple mamba blocks to obtain deep features; and (**d**) an enhanced feature fusion module that further enhances the deep features and then upsamples the features to the target resolution via pixel shuffling.

Let the input low-resolution image from an ADAS device be $I_{LR}\in {\mathbb{R}}^{3\times H\times W}$. The goal is to generate a high-resolution image $I_{SR}\in {\mathbb{R}}^{3\times rH\times rW}$ through an SR model F, and make it closer to the original high-resolution image $I_{HR}\in {\mathbb{R}}^{3\times rH\times rW}$, where r is the SR scaling factor. The overall architecture of our model, shown in Figure 2, can be expressed as:

(1) $$I_{SR}=F\left(I_{LR};\Theta \right),$$

where Θ represents the model parameters, and F consists of four main parts: the feature extraction module, the ER module, the variable mamba integration module, and the enhanced feature fusion module. Here, ‘variable’ indicates that the number of simple mamba blocks (SMB) can be adjusted. This allows the model to be configured for different computational budgets.

### 3.1. Feature Extraction Module

This module includes initial and multi-scale feature extraction, as shown in Figure 2a. Initial feature extraction is performed via a 3 × 3 convolution, represented as:

(2) $$F_{0}=f_{conv}^{3\to C}\left(I_{LR}\right),$$

in which $f_{conv}^{3\to C}$ is a convolutional layer converting the three-channel input to C-channel features. The multi-scale feature extraction process is represented as:

(3) $$F_{1}=\varepsilon_{multi}\left(F_{0}\right),$$

where $\varepsilon_{multi}$ denotes multi-scale feature extraction, composed of convolutional layers with different kernel sizes and fusion layers. The output of this module is represented as:

(4) $$F_{2}=Concat\left(f_{\max}\left(f_{conv}\left(F_{1}\right)\right),F_{0}\right).$$

Here, $f_{\max}$ denotes the LeakyReLU activation function.

### 3.2. Enhanced Residual Module

This module first fuses features extracted by n composite convolution blocks (CLC) combined with the convolutional block attention module (CBAM) attention mechanism. The depth of this module can be adjusted based on training requirements, as shown in Figure 2b. Here, $F_{2}$ is the input to this module. The CLC process, shown in Figure 2, is represented as:

(5) $$F_{3}=f_{CLC}\left(F_{2}\right)=f_{conv}\left(f_{\max}\left(f_{conv}\left(F_{2}\right)\right)\right).$$

CBAM is a lightweight attention mechanism combining channel and spatial attention, maintaining model performance while reducing computational overhead. Therefore, we embedded it into the ER module. The shallow feature output from a single ER block is then represented as:

(6) $$F_{shallow\_1}=f_{\max}\left(Concat\left(F_{2},CBAM\left(F_{3}\right)\right)\right).$$

For n ER blocks, the final shallow features are represented as:

(7) $$F_{shallow\_n}=f_{\max}\left(Concat\left(F_{shallow\_n-1},CBAM\left(f_{CLC}\left(F_{shallow\_n-1}\right)\right)\right)\right).$$

### 3.3. Variable Mamba Integration Module

CNN extracts features using local convolution kernels, with the receptive field growing linearly as the network deepens. Modeling long-range dependencies therefore requires stacking many layers, which increases computational cost and complicates optimization. Transformers model global dependencies directly via self-attention, but their complexity grows quadratically with spatial size, making them impractical for high-resolution inputs in real-time systems. Mamba, based on a selective state space model, serializes feature maps and applies a linearly complex scanning mechanism, substantially reducing computation while preserving global modeling capability.

Unlike the original mamba block, which relies solely on linear projections, our enhanced block incorporates depth-wise separable convolutions before the SSM processing. This enables local feature modulation and improves gradient flow, addressing the issue of vanishing gradients in deeper structures. To better adapt the 1D sequence modeling capability of mamba to 2D image structures, we introduce learnable positional encodings during the serialization step. This allows the model to retain spatial coherence and improves the reconstruction of structural details.

This integration module consists of m enhanced mamba blocks. The processing of each enhanced mamba block includes serialization, block processing, and deserialization, as shown in Figure 2c. First, the input feature $F_{shallow}$ undergoes serialization processes like projection and normalization, represented as:

(8) $$F_{in}=LayerNorm\left(F_{shallow}\right);F_{seq}=Liner\left(F_{in}\right).$$

Before block processing, we use depth-wise separable convolution for linear combination and the SiLU activation function to prevent gradient vanishing. This process is represented as:

(9) $$F_{ssm\_in}=SiLU\left(f_{DW-conv}\left(F_{seq}\right)\right).$$

After processing by the continuous time SSM, deserialization is performed to obtain the deep feature from a single enhanced mamba block:

(10) $$F_{deep\_i}=Concat\left(Line\left(LayerNorm\left(M\left(F_{ssm\_in}\right)\right)⊗SiLU\left(F_{seq}\right)\right),F_{shallow}\right),$$

where M denotes the SSM state model. Since we use variable-depth mamba integration blocks, for m enhanced mamba blocks, the final integrated deep feature is represented as:

(11) $$F_{deep}=Concat\left(F_{deep\_1},F_{deep\_2},...,F_{deep\_m}\right).$$

### 3.4. Enhanced Feature Fusion Module

This module enhances the deep features and performs pixel rearrangement, as shown in Figure 2d. First, a 1 × 1 convolution adjusts the channel number of the concatenated features:

(12) $$F_{fusion}=f_{conv}^{m\cdot C\to C}\left(F_{deep}\right).$$

The fused features are then fed into a simple enhanced residual (SER) block for enhancement. This module consists of two ER modules and a 1 × 1 convolution connected via a residual connection:

(13) $$F_{enhanced\_fusion}=F_{fusion\_2},$$

where $F_{fusion\_2}$ is similar to a single ER module.

Subsequently, pixel shuffling is applied to the enhanced features for upsampling:

(14) $$F_{up}=f_{pixel\_shuffle}\left(f_{conv}^{C\to C\cdot r^{2}}\left(F_{enhanced\_fusion}\right)\right).$$

Here, $f_{pixel\_shuffle}$ rearranges a feature map with $C\cdot r^{2}$ channels into C channels, increasing the spatial dimensions by a factor of r. Finally, features are fine-tuned via a custom residual structure and convolution operations to generate the final SR image:

(15) $$I_{SR}=f_{conv}^{C\to 3}f_{CLC}\left(f_{CL}\left(f_{CL}\left(F_{up}\right)\right)\right).$$

**Figure 3:**
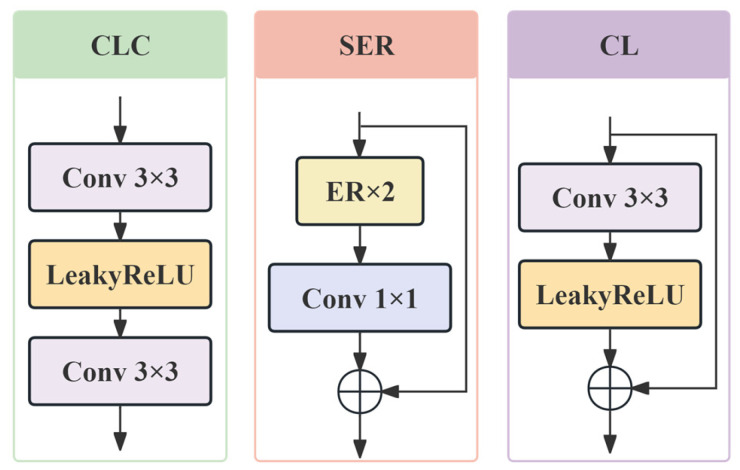
The block structure within VMESR.

Here, $f_{CL}$ denotes the custom residual structure, containing a convolutional layer and an activation function, as shown in Figure 3.

### 3.5. Loss Function

We implemented several numerical stability measures, including gradient clipping, stable weight initialization, and NaN/Inf detection during training, to ensure training stability and convergence. For the loss function, in addition to the conventional L1 loss, we incorporated perceptual loss and multi-scale loss to optimize the model. Although L1 loss can optimize pixel errors, it often results in overly smooth images and the loss of high-frequency textures. The core idea of perceptual loss is to measure the semantic similarity of images by using the high-level features of the pre-trained VGG network, rather than merely comparing pixel values. The total loss is defined as:

(16) $$L_{total}=\lambda_{1}L_{1}+\lambda_{2}L_{perceptual}+\lambda_{3}L_{multi},$$

where $L_{1}$ is the mean absolute error loss, $L_{perceptual}$ denotes the perceptual loss based on a pre-trained VGG network, and $L_{multi}$ represents the multi-scale loss. The formulations are as follows:

(17) $$L_{1}={\left\|I_{SR}-I_{HR}\right\|}_{1},$$

(18) $$L_{perceptual}=\sum_{i=1}^{N}{\left\|\Phi_{i}\left(I_{SR}\right)-\Phi_{i}\left(I_{HR}\right)\right\|}_{1},$$

(19) $$L_{multi}=\sum_{k\in \kappa }{\left\|Downsample_{k}\left(I_{SR}\right)-Downsample_{k}\left(I_{HR}\right)\right\|}_{1}.$$

Here, $\Phi_{i}\left(\cdot \right)$ denotes the feature representation of image I at the i−th layer of the pre-trained VGG network; N is the number of feature layers; and $Downsample_{k}\left(\cdot \right)$ denotes the downsampling operation with factor $\kappa =\left\{1,0.5,0.25\right\}$.

## 4. Experiments

### 4.1. Datasets

Training Datasets: DIV2K [33] contains 1000 high-resolution images, typically divided into 800 for training, 100 for validation, and 100 for testing. The images are filtered for quality, usually high-quality natural photos with rich structure, texture, and color variations. Flickr2K [34] is commonly used as a supplementary training set to DIV2K, often annotated with 2650 images. Following standard practice in the super-resolution literature, we synthesized low-resolution (LR) images by downsampling the HR images using bicubic interpolation with scaling factors of ×2, ×3, and ×4. During training, we extracted 64 × 64 pixels patches from the LR images with a stride of 32, yielding approximately 1.2 million training patches.

Test Datasets: Set5 [35] contains only five classic images with medium to high resolution, mostly portraits, animals, and close-up objects. Set14 [36] includes 14 small to medium-resolution images covering categories like people, nature, architecture, and still life. BSD100 [37] consists of 100 natural images selected from the Berkeley Segmentation Dataset, containing rich natural textures, scenery, and detail variations, making it more suitable for evaluating restoration performance on natural scenes. Urban100 [38] contains 100 high-resolution images focused on urban architecture and outdoor structures. Manga109 [39] includes 109 nonnatural images from Japanese comics, featuring a style of black and white or high-contrast lines and local color blocks. These datasets were used exclusively for testing and were not involved in any training or validation phases.

Real datasets: Cityscapes dataset [40] is designed for semantic understanding of urban street scenes, and contains 5000 finely annotated images.; we divided them into 2975 training, 500 validation, and 1525 test images, respectively. GSV-Cities dataset [41] contains around 530,000 street-view images collected from cities around the world. To reduce training cost and assess our method under constrained data conditions, we used data from five cities, including Buenos Aires, Los Angeles, Medellin, Mexico City and OSL. We divided them into 27,342 training, 4595 validation, and 14,016 test images, respectively.

### 4.2. Implementation Details

The hardware environment was Ubuntu 20.04 LTS with a GeForce RTX 3090Ti GPU. The PyTorch 1.12.1 deep learning framework was used. The input LR images were first resized to 256 × 256, and the Y channel was extracted via color space conversion. Then, the input images were cropped into several 64 × 64 patches. These patches were fed into the network for training. The training focused on comparing ×4 SR reconstruction performance with other methods. The batch size was set to 16, the training iterations to 4 × 10^5^, and the initial learning rate to 10^−4^.

### 4.3. Evaluation Metrics

Peak signal-to-noise ratio: A classical objective metric based on pixel error. It computes the mean squared error (MSE) between the reconstructed and reference images, then takes the logarithm of the ratio between the maximum pixel value and the MSE to obtain a value in decibels (dB). A higher value indicates more accurate pixel-level reconstruction.

Structural similarity index: Compares the reference and reconstructed images within local windows in terms of luminance, contrast, and structure, outputting a similarity score in the range (0, 1). A value closer to one indicates structural and perceptual quality closer to the ground truth.

Floating point operations per second: This is a measure of the computational complexity of a model or algorithm, which is the total number of floating-point operations required to complete one inference or training session.

Local attribution map (LAM): LAM [42] is a visual attribution tool for auxiliary analysis, and it typically refers to importance maps generated at the pixel or region level using attribution or saliency methods to analyze the model’s “attention” or reconstruction contribution to different local regions of the input. LAM can be used to visualize and quantify which regions are amplified, preserved, or enhanced by the model.

### 4.4. Ablation Studies

To verify the contribution of each key component in the VMESR, we designed a series of repeatable and quantifiable ablation experiments. All ablations were conducted under a scaling factor of four, with training configurations consistent with the main experiments. To ensure fairness, all replaced modules maintained a similar parameter scale and depth. Convolutional replacement modules used combinations of 3 × 3 and 1 × 1 convolutions to ensure an equivalent receptive field. Each control experiment was trained to full convergence to avoid performance bias due to early stopping.

#### 4.4.1. Effectiveness of the Mamba Module

To verify the contribution of the mamba module in modeling long-range dependencies and enhancing global contextual understanding for super-resolution, we conducted ablation experiments under the baseline configuration of *M* = 6 and *N* = 8. Specifically, we completely removed the mamba module and, in a separate experiment, replaced it with regular convolutions which had an equivalent number of parameters. This allowed us to isolate and quantify the performance gain attributable to the selective state-space modeling capability of mamba.

As shown in Table 1, completely removing the mamba module or replacing it with regular convolutions of equivalent parameters both led to significant performance degradation, with PSNR values on the Set5, Set14, BSD100, and Urban100 test sets lower than the baseline model. This indicates the irreplaceable role of the mamba module in modeling long-range dependencies and enhancing global contextual understanding, contributing significantly to overall performance improvement.

#### 4.4.2. Effectiveness of the ER Module

To verify the contribution of multi-scale fusion and attention mechanisms within the ER module for texture detail recovery, we performed ablation experiments under the baseline configuration of *M* = 6 and *N* = 8. We systematically removed the CLC block and CBAM attention mechanism from the ER module, and in a separate experiment, replaced the entire ER module with two stacked 3 × 3 convolutions and ReLU activation functions while maintaining an equivalent parameter count. These experiments aim to demonstrate whether the proposed ER module offers advantages over simple convolutional stacking in recovering fine details.

In the ER module ablation experiments, Table 2 shows that removing the entire ER module, or individually removing its key components (the CLC block or the CBAM block), all lead to performance degradation, particularly noticeable on the texture-complex Urban100 dataset. Moreover, replacing the ER module with an equivalent structure of two 3×3 convolutions plus ReLU also results in inferior performance compared to the baseline. This indicates that the feature enhancement and texture detail recovery mechanisms achieved through multi-scale fusion and attention in the ER module are effective, and its design is superior to simple convolutional stacking.

#### 4.4.3. Impact of Different Numbers of Simple Mamba Blocks (*M*)

Keeping the number of ER blocks, input/output channels, and training configuration constant, we evaluated the contribution of serialized global modeling depth to SR performance and sought the optimal trade-off under linear complexity. We tested mamba modules counts as $M\in \left\{2,4,6,8,10\right\}$.

The ablation results in Table 3 show that, with a fixed number of ER modules *N* = 8, as the number of simple mamba blocks *M* increases from 2 to 10, the PSNR performance on the BSD100 and Urban100 datasets exhibits a trend of significant initial improvement followed by saturation. Specifically, when *M* increases from 2 to 6, performance improves markedly, with BSD100 PSNR rising from 26.51 dB to 27.89 dB, indicating that increasing global modeling depth positively impacts the SR task. However, when *M* further increases to between 8 and 10, performance gains become extremely limited (only about 0.06 dB and −0.03 dB fluctuations), while the number of parameters and FLOPs continue to grow significantly. This suggests the model approaches a performance saturation point around *M* = 6. Further increasing mamba modules leads to wasted computational resources, contradicting efficient model design principles. Therefore, *M* = 6 can be considered a better trade-off point between performance and complexity.

#### 4.4.4. Impact of Different Numbers of ER Modules (*N*)

Keeping the number of simple mamba blocks, input/output channels, and training configuration constant, we evaluated the impact of parallel feature extraction to SR performance and sought the optimal trade-off under linear complexity. We tested ER modules counts as $N\in \left\{2,4,6,8,10\right\}$.

The experiments in Table 4 investigate the impact of *N* with *M* fixed at 6. Results show that as *N* increases from 2 to 10, model performance gradually improves, but the rate of improvement eventually diminishes. From *N* = 2 to *N* = 8, PSNR consistently improves, with BSD100 PSNR increasing from 27.41 dB to 27.89 dB, indicating that increasing local feature enhancement capacity helps improve reconstruction quality. However, the performance gain from *N* = 8 to *N* = 10 is negligible, only 0.03 dB, while parameters and FLOPs continue to grow linearly, suggesting *N* = 8 is near the point of diminishing returns. This result indicates that moderate stacking of ER modules can effectively enhance feature representation, but excessive stacking reduces cost-effectiveness. It is suggested that *N* = 8 serves as a reference configuration balancing computational efficiency and performance in subsequent designs.

### 4.5. Comparative Experiments

We compared the proposed method with SOTA methods, including those based on traditional CNNs, transformers, and mamba networks: SRCNN, VDSR, EDSR, MAN, CNMC, and MambaIR [43]. Experiments were conducted on five public datasets: Set5, Set14, BSD100, Urban100, and Manga109. Under scaling factors of two, three, and four we analyzed objective metrics (PSNR, SSIM, and model parameter count) to validate the effectiveness of our proposed method. Our approach includes a lightweight model VMESR-T with only 179 K parameters and a full model VMESR.

As shown in Table 5, in the experimental results across different scaling factors, VMESR demonstrates strong competitiveness across all benchmark datasets while maintaining a parameter count of only 748 K. Compared to the strongest peer method MambaIR, VMESR achieves comparable or superior PSNR/SSIM in most scenarios on Set5, Set14, BSD100, Urban100, and Manga109. Particularly under high magnification factors of ×3 and ×4, VMESR frequently achieves the best or joint-best reconstruction quality on Set5, Set14, Urban100, and Manga109, exhibiting exceptional high-frequency texture recovery capability. Furthermore, VMESR shows more stable cross-scale performance, maintaining performance consistent with SOTA levels for both low magnification and challenging ×4 scenarios. Considering both performance and parameter scale, VMESR’s performance per parameter is 1.25 times that of MambaIR and 1.19 times that of MAN, significantly higher than other methods, making it the most cost-effective solution among peer lightweight models. This fully demonstrates its effectiveness and advantageous position in the SR task.

**Figure 4:**
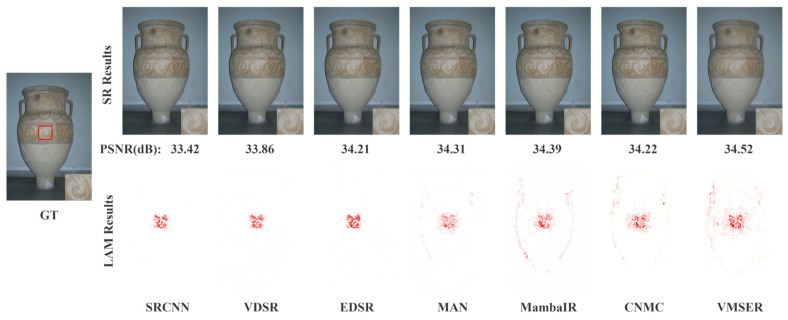
Qualitative and LAM comparison for image ‘IMG_046’ from the BSD100 dataset (×4).

**Figure 5:**
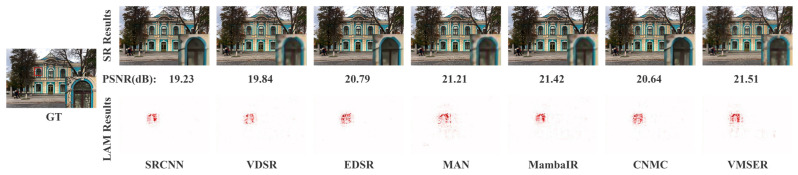
Qualitative and LAM comparison for image IMG_088 from the Urban100 dataset (×4).

As shown in Figure 4 and Figure 5, VMESR’s reconstruction results show significant advantages in image contours and details. Traditional CNN-dominated networks like SRCNN, VDSR, and EDSR yield inferior reconstruction results compared to transformer-based and mamba-based networks. In the reconstruction of the patterns on a vase and the area of a windowsill, the PSNR values of VMESR were 34.52 dB and 21.51 dB respectively, which were both the highest-scoring values. Compared with other methods, VMESR is better able to reconstruct the outline and detailed edges of the patterns on the vase, but its performance in reconstructing cracks around the patterns is relatively weak. In terms of the reconstruction details of the windowsill, VMESR clearly reconstructed the outline of the windowsill and some details of the branches, while other methods had significant deficiencies in the reconstruction of the windowsill outline and the details of the branches. For the LAM, it shows that when the model reconstructs a specific area, such as the details in the vase or the windowsill, it mainly relies on the information of which pixels are in the input image, and visualizes whether the model depends more on local details or on the global context. From the LAM results, VMESR demonstrated clearer outlines and details in the reconstructed image, including the texture on the vase and the windowsill that is obscured by the branches. For the pattern details on the vase, VMESR’s reconstruction is clearly superior to other methods, although the crack reconstruction on the vase is not distinctly better. For the windowsill reconstruction in the building scene, most methods perform poorly due to occlusion by branches, but VMESR could clearly reconstruct the outline of the windowsill. Visually, VMESR shows strong competitiveness compared to other methods.

To further verify the competitiveness of VMESR in lightweight models, in Table 6, we conducted a detailed comparison with the latest lightweight models that have similar parameter quantities on the Urban100 dataset.

From Table 6, in the ×4 SR task on the Urban100 dataset, it can be observed that VMESR achieves the highest PSNR of 27.69 dB and SSIM of 0.8302, while maintaining relatively moderate model parameters and FLOPs. MambaIR follows closely with a PSNR of 27.68 dB and the second-best SSIM of 0.8287, but at the cost of higher computational overhead and model parameters. SRCNN exhibits the lowest performance with a PSNR of 24.52 dB and SSIM of 0.7226, along with the smallest model size but relatively low FLOPs. MAN-light and MAN achieve competitive performance of PSNR with 26.70 dB and 27.26 dB, respectively, and with moderate model complexity. MambaIR-light, despite having slightly fewer parameters than MambaIR, shows a notable drop in PSNR of 26.75 dB and a SSIM of 0.8051. CNMC delivers a PSNR of 26.47 dB and SSIM of 0.7978, which is lower than MAN and VMESR, while requiring 68.5 G FLOPs. VMESR stands out as the most efficient method in terms of the trade-off between reconstruction quality and computational cost.

### 4.6. Practical Application

To further validate the performance of VMESR in the aspects of road scene understanding and application of on-board visual sensors, we conducted a ×4 magnification super-resolution quantitative comparison experiment on the real-road scene datasets Cityscapes and GSV-Cities. These two datasets cover various complex driving conditions and can more accurately reflect the performance of the model in actual on-board visual tasks. To comprehensively evaluate the performance of VMESR, we included the classic methods of SRCNN and VDSR, the SOTA methods of MAN, MambaIR and CNMC, and the lightweight models MAN-light and MambaIR-light for comparison. The experimental settings of all methods were exactly the same: the LR images were generated through double cubic downsampling, the evaluation indicators were PSNR/SSIM of the Y channel, and the preset parameters were consistent. The objective indicators are shown in Table 7.

From Table 7, it can be seen that different super-resolution methods have significant trade-offs in terms of performance, efficiency, and model complexity. VMESR achieved the highest PSNR of 31.86 dB and SSIM of 0.8991 on the Cityscapes dataset, and ranked second on the GSV-Cities dataset with a PSNR of 30.11 dB and SSIM of 0.9023. At the same time, its inference time only required 23 ms, demonstrating excellent efficiency and performance balance, making it a highly competitive choice for practical applications. MambaIR, although topping the GSV-Cities dataset with a PSNR of 30.21 dB and SSIM of 0.9064, had the largest parameter of 927 K and the longest cost time of 67 ms, making it more suitable for scenarios with extremely high precision but insensitive to time. The MAN series and MambaIR-light models, although displaying good performance, had higher time costs; traditional SRCNN and VDSR, although extremely fast, had significantly lower image quality compared to modern methods. Therefore, balancing image quality and speed, VMESR was the best choice; if pursuing ultimate performance, one could consider MambaIR.

We collaborated with Eteck Automotive Electronics company to capture real driving scenes through the front camera of the car for testing. This data was then used to test the effectiveness of our SR reconstruction algorithm on authentic, real-world images

**Figure 6:**
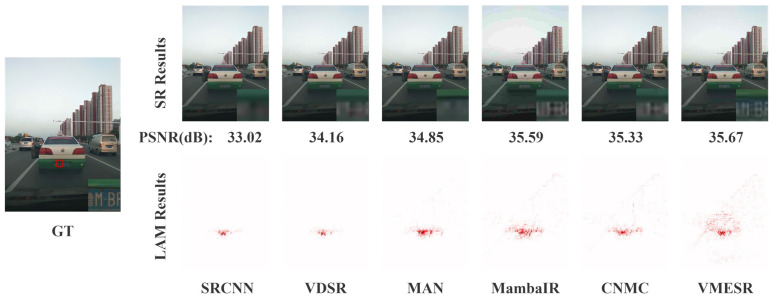
Qualitative and LAM comparison for a leading vehicle in a real-world scene (×4).

**Figure 7:**
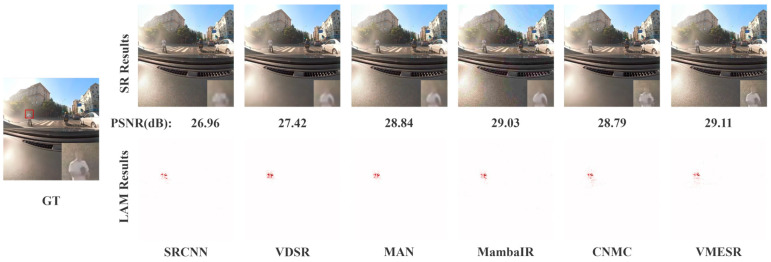
Qualitative and LAM comparison for a pedestrian in a real-world scene (×4).

The experiments of visualization are essentially zero-shot tests, which can truly reflect the performance of the model when it is deployed in practice. From Figure 6 and Figure 7, it can be observed that in real automotive scenarios, VMESR significantly outperforms other advanced methods in reconstructing critical visual information, such as the license plates of the vehicles ahead and the texture of the pedestrians’ clothing. In the license plate area, VMESR successfully restored clear outlines of the numbers and letters, while the results of MAN and MambaIR were significantly blurry, and achieved a PSNR of 35.67 dB, outperforming all other methods. In the pedestrian and clothing scenarios, VMESR accurately reconstructed the fine textures around the subjects; although CNMC reconstructed the content of this area, it was 0.32 dB lower in the PSNR index than VMESR, and other methods presented a roughly blocky distribution and failed to reconstruct this area of pedestrians and clothing well. Further quantitative analysis showed that VMESR required only 23 ms to process a single frame in real-world scenes, meeting the real-time requirements of automotive systems. Experiments prove that VMESR maintains stable performance under practical interfering factors such as complex lighting and motion blur. Its lightweight design allows direct deployment on existing vehicle-mounted chip platforms, providing high-resolution visual input for ADAS and effectively improving the accuracy of object detection and scene understanding.

## 5. Conclusions

We proposed VMESR, a lightweight image super-resolution framework based on a variable mamba state-space model, which aims to meet the dual demands of real-time performance and high-quality reconstruction for autonomous driving and automotive vision systems. VMESR achieves efficient global context modeling and fine texture recovery through multi-scale feature extraction, ER modules, CBAM attention mechanisms, and variable-depth mamba modules. Experimental results on multiple public benchmarks demonstrate that VMESR achieves comparable or even superior SR performance to existing SOTA methods while using an extremely small number of parameters. Moreover, in real vehicle-mounted camera scenarios, VMESR significantly enhances image quality under challenging conditions like harsh weather and low illumination, delivering stable performance gains for downstream autonomous driving perception tasks. VMESR combines efficiency, robustness, and good effectiveness, offering a feasible and valuable research path for future embedded automotive vision enhancement systems and providing new insights into designing SR models in low-energy consumption scenarios. In the next stage, we plan to conduct end-to-end evaluation with the Eteck Automotive Electronics company on real driving datasets, integrating VMESR into the object detection and semantic segmentation processes, and systematically analyzing its performance gains under different degradation conditions. We believe that such a systematic evaluation will more comprehensively reveal the contribution of super-resolution to autonomous driving perception.

## Figures and Tables

**Table 1:** Ablation studies for different design choices of mamba module. Evaluation metric is PSNR (dB); higher is better.

Settings	Set5	Set14	BSD100	Urban100
W/O mamba module	32.21	27.97	27.14	25.94
Replace with Conv	32.42	28.15	27.31	26.24
Baseline	33.11	29.24	27.89	27.69

**Table 2:** Ablation studies for different design choices of ER module. Evaluation metric is PSNR (dB); higher is better.

Settings	Set5	Set14	BSD100	Urban100
W/O ER module	32.15	27.85	27.09	25.87
W/O CLC block	32.37	29.02	27.45	27.12
W/O CBAM block	32.42	29.09	27.66	27.24
Replace with Conv	32.39	28.89	27.11	26.57
Baseline	33.11	29.24	27.89	27.69

**Table 3:** Ablation studies for different numbers of mamba blocks (*M*).

Settings	BSD100	Urban100	Parameters	FLOPs
	(dB)	(dB)	(K)	(G)
*M* = 2, *N* = 8	26.51	26.84	612	32.3
*M* = 4, *N* = 8	27.41	27.27	675	38.5
*M* = 6, *N* = 8	27.89	27.69	748	41.2
*M* = 8, *N* = 8	27.95	27.74	822	45.1
*M* = 10, *N* = 8	27.92	27.71	924	52.1

**Table 4:** Ablation studies for different numbers of ER modules (*N*).

Settings	BSD100	Urban100	Parameters	FLOPs
	(dB)	(dB)	(K)	(G)
*N* = 2, *M* = 6	27.41	27.12	637	34.3
*N* = 4, *M* = 6	27.62	27.44	674	38.5
*N* = 6, *M* = 6	27.75	27.54	711	39.6
*N* = 8, *M* = 6	27.89	27.69	748	41.2
*N* = 10, *M* = 6	27.92	27.68	785	43.6

**Table 5:** Quantitative results of different methods on Set5, Set14, BSD100, Urban100 and Manga109 datasets under scaling factors of two, three and four.

Methods	Scale	Parameters (K)	Set5	Set14	BSD100	Urban100	Manga109
			PSNR/SSIM	PSNR/SSIM	PSNR/SSIM	PSNR/SSIM	PSNR/SSIM
SRCNN	×2	**57**	33.68/0.9304	32.45/0.9067	31.36/0.8879	29.51/0.8946	35.72/0.9680
VDSR	×2	665	37.53/0.9587	33.05/0.9127	31.90/0.8960	30.77/0.9141	37.16/0.9740
EDSR	×2	43,090	38.11/0.9602	33.92/0.9195	32.32/0.9013	32.93/0.9351	39.10/0.9773
MAN	×2	870	38.42/0.9622	34.40/0.9242	32.53/0.9043	33.73/0.9422	40.02/0.9801
MambaIR	×2	905	**38.57**/0.9627	**34.67**/**0.9261**	32.58/0.9048	**34.15**/0.9446	**40.28**/0.9806
CNMC	×2	769	38.14/0.9610	33.84/0.9203	32.29/0.9011	32.74/0.9337	39.13/0.9775
VMESR-T	×2	169	38.11/0.9621	33.85/0.9213	32.15/0.9002	32.88/0.9307	39.21/0.9645
VMESR	×2	721	38.44/**0.9631**	34.52/0.9255	**32.61**/**0.9077**	**33.87**/**0.9447**	**40.21**/0.9812
SRCNN	×3	**57**	32.72/0.9090	29.29/0.8215	28.41/0.7863	26.24/0.7991	30.48/0.9117
VDSR	×3	665	33.66/0.9213	29.77/0.8318	28.83/0.7976	27.14/0.8279	32.01/0.9340
EDSR	×3	43,090	34.65/0.9280	30.52/0.8462	29.25/0.8093	28.80/0.8653	34.17/0.9476
MAN	×3	870	34.91/0.9312	30.88/0.8514	29.43/0.8138	29.52/0.8782	35.06/0.9526
MambaIR	×3	912	35.08/0.9323	30.99/**0.8536**	**29.51**/**0.8157**	**29.93**/**0.8841**	35.43/0.9546
CNMC	×3	775	34.57/0.9285	30.52/0.8456	29.19/0.8080	28.61/0.8614	34.02/0.9471
VMESR-T	×3	172	34.54/0.9274	30.42/0.8454	29.11/0.8002	28.81/0.8686	34.25/0.9489
VMESR	×3	730	**35.12**/**0.9328**	**31.03**/0.8534	29.49/0.8149	29.62/0.8712	**35.44**/**0.9553**
SRCNN	×4	**57**	30.48/0.8628	27.50/0.7513	26.90/0.7103	24.52/0.7226	27.66/0.8580
VDSR	×4	665	31.35/0.8838	28.02/0.7678	27.29/0.7252	25.18/0.7525	28.82/0.8860
EDSR	×4	43,090	32.46/0.8968	28.80/0.7876	27.71/0.7420	26.64/0.8033	31.02/0.9148
MAN	×4	870	32.81/0.9024	29.07/0.7934	27.90/0.7477	27.26/0.8197	31.92/0.9230
MambaIR	×4	924	33.03/0.9046	29.20/0.7961	**27.98**/**0.7503**	27.68/0.8287	32.32/**0.9272**
CNMC	×4	782	32.39/0.8975	28.73/0.7851	27.68/0.7402	26.47/0.7978	30.96/0.9135
VMESR-T	×4	179	32.09/0.8590	28.73/0.7855	27.41/0.7391	26.17/0.7855	31.77/0.9184
VMESR	×4	748	**33.11**/**0.9122**	**29.24**/**0.8084**	27.89/0.7492	**27.69**/**0.8302**	**32.35**/0.9267

The optimal PSNR and SSIM results are displayed in bold, while the suboptimal results are underlined.

**Table 6:** Performance results of different methods on the Urban100 dataset under a scaling factor of four.

Methods	PSNR	SSIM	Params (K)	FLOPs (G)
SRCNN	24.52	0.7226	57	5.1
VDSR	25.18	0.7525	665	16.2
MAN-light	26.70	0.8052	840	47.1
MAN	27.26	0.8197	870	48.6
MambaIR-light	26.75	0.8051	859	88.0
MambaIR	27.68	0.8287	924	102.4
CNMC	26.47	0.7978	782	68.5
VMESR	27.69	0.8302	748	41.2

**Table 7:** Performance results of different methods on the Cityscapes dataset and GSV-Cities dataset under a scaling factor of 4.

Methods	Parameters (K)	Cityscapes	GSV-Cities	Time (ms)
		PSNR/SSIM	PSNR/SSIM	
SRCNN	57	26.78/0.7546	26.20/0.8427	4
VDSR	665	29.00/0.8196	27.39/0.8737	7
MAN-light	840	31.02/0.8825	29.13/0.8912	28
MAN	870	31.75/0.8902	29.64/0.9034	39
MambaIR-light	859	31.15/0.8853	29.28/0.8945	45
MambaIR	927	31.84/0.8954	30.21/0.9064	67
CNMC	782	30.21/0.8698	28.25/0.8841	51
VMESR	748	31.86/0.8991	30.11/0.9023	23

## Data Availability

The original contributions presented in this study are included in the article. Further inquiries can be directed to the corresponding author.
